# Association between risk of asthma and gene polymorphisms in CHI3L1 and CHIA: a systematic meta-analysis

**DOI:** 10.1186/s12890-017-0515-2

**Published:** 2017-12-12

**Authors:** Yanting Zhu, Xin Yan, Cui Zhai, Lan Yang, Manxiang Li

**Affiliations:** Department of Respiratory Medicine, The First Affiliated Hospital of Xi’an Jiaotong University, No.277, West Yanta Road, Xi’an, 710061 Shaanxi People’s Republic of China

**Keywords:** Asthma, Gene polymorphism, CHI3L1, CHIA, Meta-analysis

## Abstract

**Background:**

Previous studies have indicated that chitinase 3-like 1 (CHI3L1) gene rs4950928 polymorphism and acidic mammalian chitinase (AMCase or CHIA) gene rs10494132 polymorphism are associated with the risk of asthma. However, the results are inconsistent because of small sample size and varied ethnicity and age in studies. Therefore, a systematic meta-analysis was important to clarify the effect of CHI3L1 rs4950928 polymorphism and CHIA rs10494132 variant on asthma risk.

**Methods:**

An electronic literature search was conducted to identify all the eligible studies. Odds ratios (ORs) with 95% confidence intervals (CIs) were calculated and sensitivity analysis as well as publication bias were assessed to investigate the associations. All statistical analyses were performed using STATA 12.0.

**Results:**

Eight published articles with 10 case-control studies were included, 5 studies were of CHI3L1 rs4950928 polymorphism and another 5 studies involved CHIA rs10494132 polymorphism. Overall, no significant association was found between CHI3L1 polymorphism and asthma susceptibility. After stratified according to ethnicity, CHI3L1 rs4950928 variant was associated with decreased asthma risk in Caucasians (GG + GC vs. CC: OR = 0.621, 95% CI = 0.484–0.797, *P* = 0.000; GC vs. CC: OR = 0.612, 95% CI = 0.470–0.796, *P* = 0.000; G vs. C: OR = 0.696, 95% CI = 0.567–0.856, *P* = 0.001). When stratified population by age, there was no association in children under all genetic models. As for CHIA rs10494132 polymorphism, no evidence of association between CHIA rs10494132 polymorphism and asthma risk was identified. Furthermore, subgroup analysis by ethnicity revealed a positive correlation between CHIA rs10494132 polymorphism and asthma risk among Asians (TT vs. TC + CC: OR = 1.476, 95% CI = 1.071–2.032, *P* = 0.017; T vs. C: OR = 1.326, 95% CI = 1.024–1.717, *P* = 0.032). Additionally, in the subgroup analysis conducted according to age, CHIA rs10494132 variant was also found to be associated with the increased risk of asthma in children (TT vs. TC + CC: OR = 1.472, 95% CI = 1.067–2.030, *P* = 0.019; T vs. C: OR = 1.320, 95% CI = 1.016–1.713, *P* = 0.037).

**Conclusions:**

The G allele of CHI3L1 rs4950928 might be a protective factor against the development of asthma. However, the rs10494132 polymorphism of CHIA might be a risk factor for asthma.

## Background

Asthma is a chronic inflammatory respiratory disease that is characterized by airway hyper-reactivity to some environmental stimuli, reversible airway obstruction, and gradually appeared airway structural remodeling [1]. Although the exact etiology of asthma remains uncertain, it has become evident that a combination of genetic predisposition and environmental exposures plays a vital role in pathogenesis of asthma [2–4]. Recently, with numerous advances in genetic research, many genetic variants regulating the expression of proteins involved in the development of asthma have been found to be related to susceptibility to this disease, including interleukin-17F (IL-17F), toll-like receptor 4 (TLR4), and the genes encoding chitinase 3–like 1 (CHI3L1) and acidic mammalian chitinase (AMCase or CHIA) [5–8].

Chitin is an abundant, naturally occurring polysaccharide functioning as a major structural polymer in many lower life forms as well as an important nutritional source for many organisms, which triggers allergies or asthma attacks in people [9–11]. The chitinase family has three members: two of which are the true chitinases—acidic mammalian chitinase and chitotriosidase (CHIT); the other is the structurally related chitinase-like protein YKL-40 [12–14]. It has been reported that chitinase and chitinase-like proteins are highly expressed in lungs and serum of asthma patients [12], which are also found to regulate TH2 immune response and mediate airway inflammation and airway remodeling in mouse asthmatic models [15, 16]. YKL-40 is encoded by the chitinase 3-like 1 gene (CHI3L1), which is 10 exons long and is located on chromosome 1q31-q32 [17]. Genetic studies have demonstrated that the variants of CHI3L1 may contribute to the pathogenesis of asthma [7, 18]. A Genome-wide Association Study (GWAS) has identified that the rs4950928 polymorphism in the CHI3L1 gene is associated with the risk of asthma, circulating YKL-40 level and pulmonary function [18]. The gene for CHIA is present on chromosome 1q13.1–21.3 and contains 12 exons [13]. Recent genetic study has described that several CHIA polymorphisms is related to asthma risk in a German pediatric population [19].

Although several genetic studies have reported the correlation between CHI3L1 rs4950928 polymorphism or CHIA rs10494132 variant and the risk of asthma [7, 8, 18, 20], these results were controversial and inconclusive since they are performed in an individual medical center with small sample size and in different ethnicities. Therefore, it is important to perform a meta-analysis of all eligible studies to clarify the effects of CHI3L1 rs4950928 polymorphism and CHIA rs10494132 variant on risk of asthma.

## Methods

### Literature search strategy

The systematic electronic databases of PubMed, Embase, Web of Science, China National Knowledge Infrastructure (CNKI) and Wanfang Database were comprehensively searched for all the eligible studies published up to May 2017 using the search terms: (“chitinase 3–like 1” or “CHI3L1”), (“single nucleotide polymorphism” or “SNP” or “polymorphism” or “variation” or “mutation”) and (“asthma” or “asthmatic”) or (“acidic mammalian chitinase” or “CHIA”), (“single nucleotide polymorphism” or “SNP” or “polymorphism” or “variation” or “mutation”) and (“asthma” or “asthmatic”). Besides, the reference lists of all selected articles were manually searched for further potentially eligible articles. No restrictions with regard to language, population, publication date, or type of report were imposed; and unpublished data were excluded.

### Inclusion and exclusion criteria

All the potential studies were independently selected by two reviewers (Yanting Zhu and Xin Yan) according to the following inclusion and exclusion criteria. Inclusion criteria in this meta-analysis were: (1) studies that were using a case-control design; (2) studies that evaluated the correlation between CHI3L1 rs4950928 polymorphism or CHIA rs10494132 polymorphism and risk of asthma; (3) studies that provided sufficient data on the genotypic and allelic distributions of CHI3L1 rs4950928 polymorphism or CHIA rs10494132 polymorphism for estimating the odds ratios (ORs) and its corresponding 95% confidence intervals (CIs). Exclusion criteria were as follow: (1) studies without the control groups; (2) studies with no available data reported; (3) studies with inappropriate article types (reviews, case reports, letters, or abstracts); (4) studies with duplicated data. For overlapping studies, the largest or most recent publication was selected.

### Data extraction

The information of each eligible study was independently extracted by two investigators (Cui Zhai and Lan Yang). The following data were extracted: first author, year of publication, country, ethnicity, age, number of cases and controls, genotyping method and genotype distribution in cases and controls. Discrepancies were resolved by discussion or additional assessment by a third author (Manxiang Li), and consensus was achieved on each item.

### Subgroup criteria

Subgroup analyses were performed according to the ethnicity and age. Ethnicity is determined by the descent of all the subjects in each eligible study. For CHI3L1 rs4950928 polymorphism, three studies of European descent from European countries Sweden, Germany and USA, were enrolled and belonged to Caucasian ethnicity. One study of Asian descent came from China, which belonged to Asian ethnicity. Another study of African descent from Mauritius belonged to African ethnicity. As for CHIA rs10494132 polymorphism, three studies of Asian descent, belonged to Asian ethnicity, came from China and were enrolled in this study. Another two studies of European descent came from North India and belonged to Caucasian ethnicity. As depicted in Table 1, for CHI3L1 rs4950928 polymorphism, three studies with an age of 0–16 years were considered as children group. One study with a range of 18–72 years was adults group. Another study range from 7 to 74 was mixture group. As for CHIA rs10494132 polymorphism, two studies range from 5 to 14 years were considered as children group. Another two studies with a range of 18–49 years were adults group. Asthma and genetic susceptibility are different in 0–4 years compared to early school age and high school students. However, we could not perform subgroup analysis in children group according to different stages (preschool age, early school age and high school age) duo to insufficiency of relevant studies in literature. Gender is also an important factor for the heterogeneity. While, it was impossible to perform subgroup analysis according to gender since original data in the eligible studies were insufficient.

**Table 1:** Main characteristics of all eligible studies in this meta-analysis

Author	Year	Country	Ethnicity	Age group	Cases	Controls	Genotyping method
rs4950928							
Li [21]	2015	China	Asian	Children	316	297	Mass Array
James [7]	2016	Sweden	Caucasian	Adults	111	57	TaqMan
Ober [18]	2008	USA	Caucasian	Children	344	294	TaqMan
Ober [18]	2008	USA	Caucasian	Mixed	99	197	TaqMan
Ramphul [20]	2015	Mauritius	African	Children	192	189	TaqMan
rs10494132							
Chatterjee [8]	2008	India	Caucasian	Adults	270	292	EMSAs
Chatterjee [8]	2008	India	Caucasian	Adults	150	101	EMSAs
Huang [23]	2013	China	Asian	Children	158	198	Mass Array
Chen [22]	2014	China	Asian	Adults	150	101	Mass Array
Shao [24]	2017	China	Asian	Children	68	52	Mass Array

*NA* Not available, *EMSAs* Electrophoretic mobility shift assays

### Statistical analysis

This meta-analysis was performed using STATA version 12.0 software (Stata Corporation, College Station, Texas, USA). The Hardy-Weinberg equilibrium (HWE) for the genotype frequencies was evaluated only in controls using Chi-squared test to check whether samples in eligible studies were from a large population in HWE and whether the sample bias existed. Samples in cases may show departure from HWE since the biases existed in case groups, such as age, the ratio of gender and so on. *P* < 0.05 was considered statistically significant in HWE testing. The between-study heterogeneity was assessed by Chi-square-based Q test and *I* ^2^ statistics. The random-effect model was adopted for analysis, if there was statistical heterogeneity (*P* < 0.10). Otherwise, the fixed-effect model was applied for analysis as the pooling method. The pooled ORs and its corresponding 95%CIs were calculated to evaluate the associations of CHI3L1 rs4950928 polymorphism and CHIA rs10494132 polymorphism with asthma risk under five genetic models: recessive model (CHI3L1 rs4950928: GG vs. GC + CC; CHIA rs10494132: TT vs. TC + CC); dominant model (CHI3L1 rs4950928: GG + GC vs. CC; CHIA rs10494132: TT + TC vs. CC); codominant model (CHI3L1 rs4950928: GC vs. CC; CHIA rs10494132: TC vs. CC); homozygote model (CHI3L1 rs4950928: GG vs. CC; CHIA rs10494132: TT vs. CC); allele model (CHI3L1 rs4950928: G vs. C; rs10494132: T vs. C). Z-test was performed to assess the significance of the pooled ORs, and *P* < 0.05 considered statistically significant. Publication bias was assessed by Begg’s test and Egger’s test and *P* < 0.05 was considered significant publication bias.

## Results

### Study characteristics

**Fig. 1:**
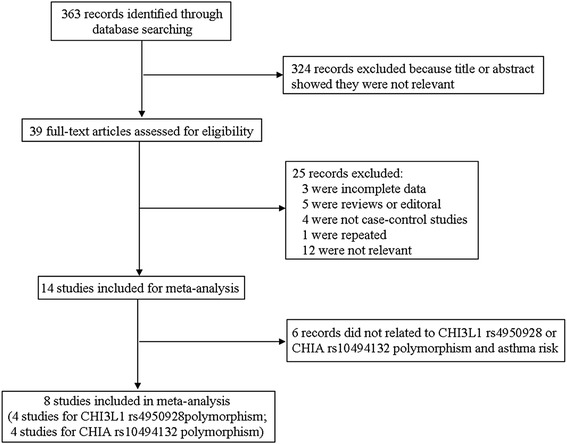
Flowchart of study selection procedure

The literature retrieval process was presented in Fig. 1. In total, 363 articles were identified through the initial search of databases. After a systematical literature search based on the inclusion criteria, 10 case-control studies from 8 published articles containing 1858 cases and 1778 controls were identified for this meta-analysis to investigate the relations of CHI3L1 and CHIA variants with asthma risk [7, 8, 18, 20–24]. Among the selected studies, 5 studies in 4 eligible publications [7, 18, 20, 21] were conducted for evaluating CHI3L1 rs4950928 polymorphism with a total of 1062 cases and 1034 controls enrolled; 5 studies containing 796 cases and 744 controls were performed for investigating CHIA rs10494132 polymorphism in 4 published articles [8, 22–24]. The main characteristics of each study were shown in Table 1. For CHI3L1 rs4950928 polymorphism, 3 studies [7, 18] were carried out among Caucasians, 1 study [21] was conducted in Asians and another one [20] was conducted in African subjects in terms of ethnicity. 3 studies on the CHI3L1 rs4950928 polymorphism were conducted in children [18, 20, 21]. Only 1 study was performed in adults [7] and the rest was performed in mixture [18]. As for CHIA rs10494132 polymorphism, there were 3 studies [22–24] of Asian ethnicity and 2 studies [8] of Caucasian ethnicity. 3 studies [8, 23, 24] were performed in children and the rest [8, 22] were conducted in adults. Genotype distributions and HWE examination results were exhibited in Table 2. The genotypes of CHI3L1 rs4950928 polymorphism in the control groups were in agreement with HWE (*P* > 0.05). As for CHIA rs10494132 polymorphism, HWE deviation existed in control of 1 eligible study [24].

**Table 2:** Distributions of CHI3L1 rs4950928 and CHIA rs10494132 polymorphisms among asthmatic patients and controls

Study	Cases			Controls			*P* for HWE
rs4950928	GG	GC	CC	GG	GC	CC	
Li [21]	13	111	192	9	71	217	0.288
James [7]	8	34	69	3	14	40	0.254
Ober [18]	17	100	227	24	120	150	1.000
Ober [18]	5	25	69	7	80	111	0.103
Ramphul [20]	6	64	122	5	63	121	0.339
rs10494132	TT	TC	CC	TT	TC	CC	
Chatterjee [8]	150	85	30	166	97	24	0.075
Chatterjee [8]	91	43	14	55	38	6	0.868
Huang [23]	130	26	2	138	50	10	0.063
Chen [22]	91	43	16	55	38	8	0.691
Shao [24]	30	22	16	23	17	12	0.023

*HWE* Hardy- Weinberg equilibrium

### Meta-analysis and subgroup analysis

The genetic effects of CHI3L1 rs4950928 polymorphism and CHIA rs10494132 variant on asthma risk were presented in Table 3.

**Table 3:** Meta-analysis of CHI3L1 rs4950928 and CHIA rs10494132 polymorphisms and asthma

Subgroup	Genetic model	Genotype/Allele	Type of model	Heterogeneity		Test of Association		
				I^2^	*P*_het_	OR	95% CI	*P*_meta_
rs4950928								
Overall	Recessive model	GG vs. GC + CC	F	0.00%	0.430	0.946	0.626–1.428	0.791
	Dominant model	GG + GC vs. CC	R	86.70%	0.000	0.917	0.767–1.097	0.342
	Codominant model	GC vs. CC	R	85.80%	0.000	0.915	0.759–1.103	0.351
	Homozygote model	GG vs. CC	F	39.10%	0.160	0.889	0.588–1.346	0.579
	Allele model	G vs. C	R	84.90%	0.000	0.933	0.802–1.085	0.369
Caucasians	Recessive model	GG vs. GC + CC	F	21.40%	0.128	0.794	0.475–1.327	0.378
	Dominant model	GG + GC vs. CC	R	70.20%	0.035	0.621	0.484–0.797	0.000
	Codominant model	GC vs. CC	R	65.70%	0.054	0.612	0.470–0.796	0.000
	Homozygote model	GGvs. CC	F	40.40%	0.187	0.675	0.402–1.134	0.138
	Allele model	G vs. C	R	68.70%	0.041	0.696	0.567–0.856	0.001
Children	Recessive model	GG vs. GC + CC	F	28.00%	0.249	0.844	0.529-1.346	0.476
	Dominant model	GG + GC vs. CC	R	91.90%	0.000	0.955	0.782–1.166	0.651
	Codominant model	GC + CC	R	90.90%	0.000	0.970	0.788-1.196	0.777
	Homozygote model	GG vs. CC	R	64.00%	0.062	0.797	0.499-1.275	0.345
	Allele model	G vs. C	R	91.30%	0.000	0.946	0.799–1.120	0.522
rs10494132								
Overall	Recessive model	TT vs. TC + CC	F	35.80%	0.182	1.212	0.981–1.497	0.075
	Dominant model	TT + TC vs. CC	F	21.80%	0.276	0.857	0.597–1.230	0.403
	Codominant model	TC vs. CC	F	0.00%	0.455	0.748	0.504–1.109	0.149
	Homozygote model	TT vs. CC	F	24.70%	0.257	0.916	0.630–1.331	0.646
	Allele model	T vs. C	R	57.90%	0.050	1.094	0.924–1.297	0.298
Asians	Recessive model	TT vs. TC + CC	F	29.80%	0.240	1.476	1.071–2.032	0.017
	Dominant model	TT + TC vs. CC	F	48.10%	0.145	1.125	0.654–1.933	0.671
	Codominant model	TC vs. CC	F	24.60%	0.266	0.926	0.507–1.691	0.802
	Homozygote model	TT vs. CC	F	48.90%	0.141	1.249	0.709-2.199	0.441
	Allele model	T vs. C	R	64.00%	0.062	1.326	1.024–1.717	0.032
Children	Recessive model	TT vs. TC + CC	F	30.60%	0.263	1.472	1.067–2.030	0.019
	Dominant model	TT + TC vs. CC	F	53.00%	0.119	1.101	0.629–1.927	0.737
	Codominant model	TC vs. CC	F	34.70%	0.216	0.903	0.485–1.681	0.748
	Homozygote model	TT vs. CC	F	53.30%	0.117	1.217	0.679–2.184	0.510
	Allele model	T vs. C	R	64.90%	0.058	1.320	1.016–1.713	0.037

*OR* Odds ratio, *CI* Confidence interval, *P*_*het*_ *P*-value of heterogeneity test, *P*_*meta*_ P-value of pooled effect, *R* Random effect model, *F* Fixed effect model

**Fig. 2:**
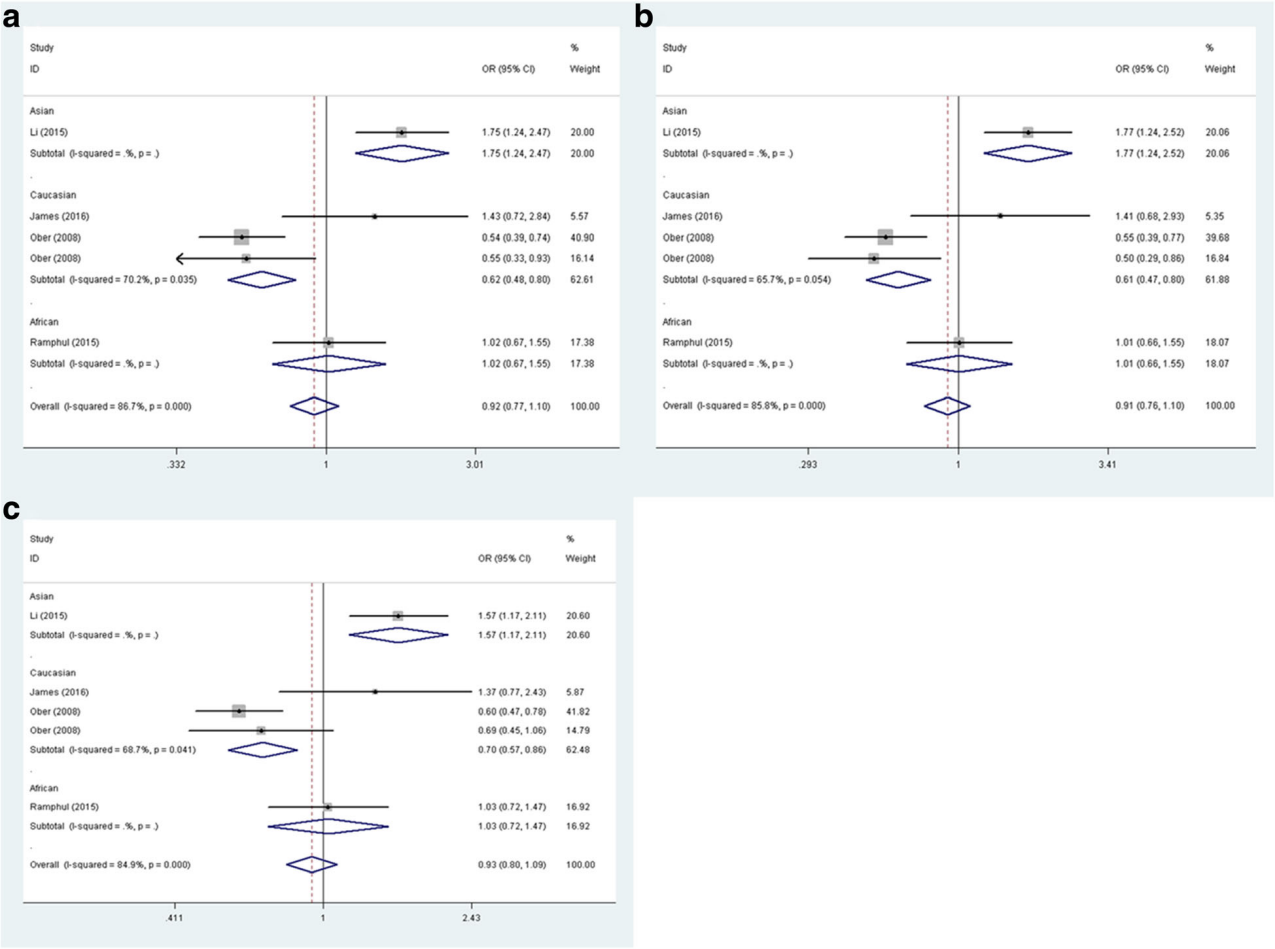
Forest plot of the association between CHI3L1 rs4950928 polymorphism and asthma risk by ethnicity stratification under (**a**) the dominant model (GG + GC vs. CC), (**b**) the codominant model (GC vs. CC) and (**c**) the allele model (G vs. C)

For CHI3L1 rs4950928 polymorphism, there was no statistically significant association observed under the following genetic models: recessive model (GG vs. GC + CC: OR = 0.946, 95% CI = 0.626–1.428, *P* = 0.791); dominant model (GG + GC vs. CC: OR = 0.917, 95% CI = 0.767–1.097, *P* = 0.342); codominant model (GC vs. CC: OR = 0.915, 95% CI = 0.759–1.103, *P* = 0.351); homozygote model (GG vs. CC: OR = 0.889, 95% CI = 0.588–1.346, *P* = 0.579) and allele model (G vs. C: OR = 0.933, 95% CI = 0.802–1.058, *P* = 0.369). After categorizing included studies into different subgroups according to the ethnicity of study population, results indicated that CHI3L1 rs4950928 variant was associated with decreased asthma risk among Caucasians under the dominant model (GG + GC vs. CC: OR = 0.621, 95% CI = 0.484–0.797, *P* = 0.000), the codominant model (GC vs. CC: OR = 0.612, 95% CI = 0.470–0.796, P = 0.000) and the allele model (G vs. C: OR = 0.696, 95% CI = 0.567–0.856, *P* = 0.001) (Fig. 2). While, no increased or reduced risk of asthma was examined among Caucasians under other genetic models (recessive model, GG vs. GC + CC: OR = 0.794, 95% CI = 0.475–1.327, *P* = 0.378 and homozygote model, GG vs. CC: OR = 0.675, 95% CI = 0.402–1.134, *P* = 0.138). No subgroup analysis was performed in Asians and Africans due to only 1 study included in Asians and Africans. When stratified population by age, no statistically significant association was found in children under all genetic models. We could not perform analysis in adults since only one study was included.

**Fig. 3:**
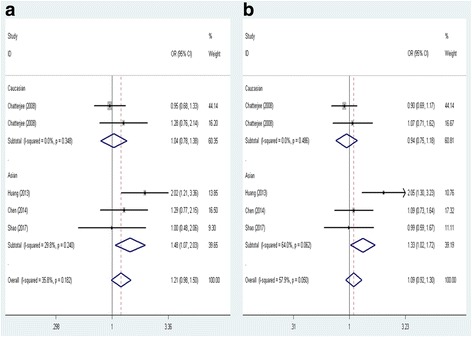
Forest plot of the association between CHIA rs10494132 polymorphism and asthma risk stratified by ethnicity under (**a**) the recessive model (TT vs. TC + CC) and (**b**) the allele model (T vs. C)

**Fig. 4:**
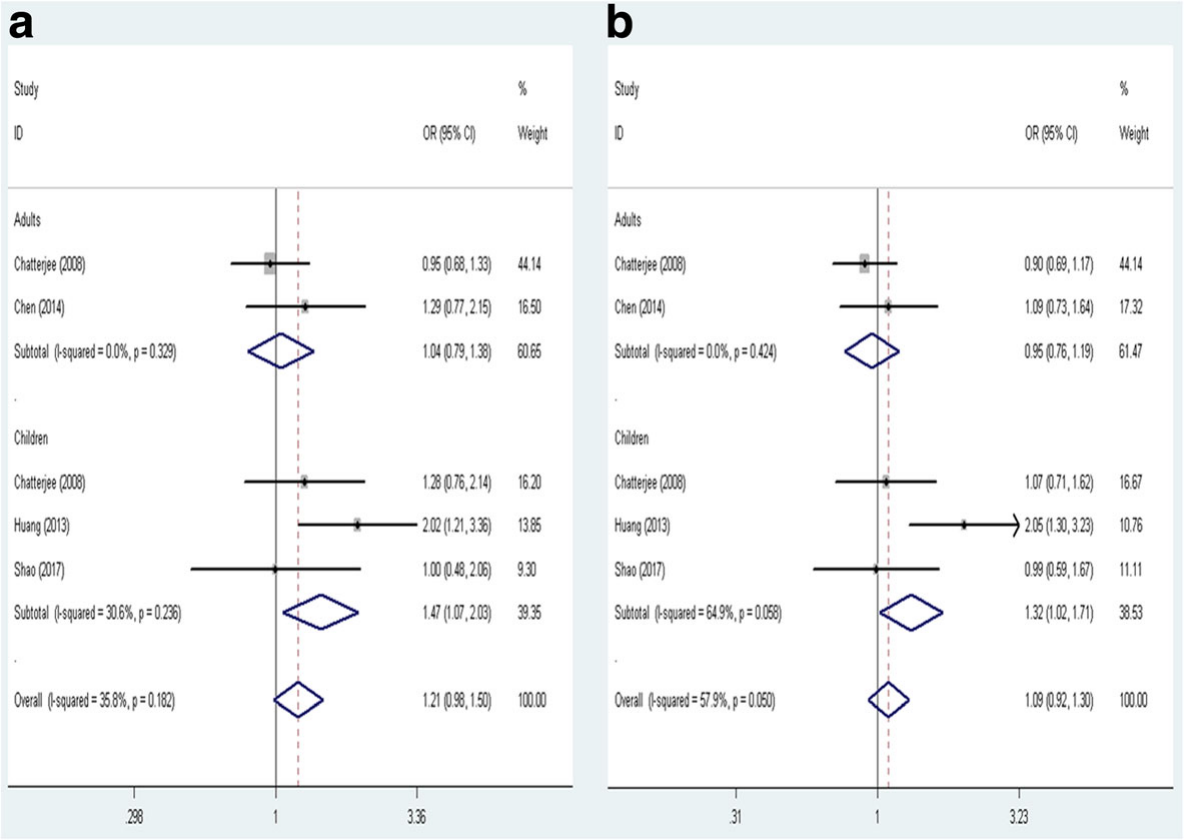
Forest plot of the association between CHIA rs10494132 polymorphism and asthma risk by age stratification under (**a**) the recessive model (TT vs. TC + CC) and (**b**) the allele model (T vs. C)

As for CHIA rs10494132 polymorphism, results based on all included studies provided no evidence of association between CHIA rs10494132 polymorphism and asthma risk in any genetic models in the overall population (recessive model, TT vs. TC + CC: OR = 1.212, 95% CI = 0.981–1.497, *P* = 0.075; dominant model, TT + TC vs. CC: OR = 0.857, 95% CI = 0.597–1.230, *P* = 0.403; codominant model, TC vs. CC: OR = 0.748, 95% CI = 0.504–1.109, *P* = 0.149; homozygote model, TT vs. CC: OR = 0.916, 95% CI = 0.630–1.331, *P* = 0.646 and allele model, T vs. C: OR = 1.094, 95% CI = 0.924–1.297, *P* = 0.298). In the subgroup analysis done on the basis of ethnicity, a positive correlation was found between CHIA rs10494132 polymorphism and asthma risk among Asians under the recessive model (TT vs. TC + CC: OR = 1.476, 95% CI = 1.071–2.032, *P* = 0.017) and the allele model (T vs. C: OR = 1.326, 95% CI = 1.024–1.717, *P* = 0.032) (Fig. 3). However, no significant association was observed under other genetic models (dominant model, TT + TC vs. CC: OR = 1.125, 95% CI = 0.654–1.933, *P* = 0.671; codominant model, TC vs. CC: OR = 0.926, 95% CI = 0.507–1.691, *P* = 0.802 and homozygote model, TT vs. CC: OR = 1.249, 95% CI = 0.709–2.199, *P* = 0.441). No subgroup analysis was performed in Caucasians due to limited studies included. Additionally, in the subgroup analysis conducted according to age, CHIA rs10494132 variant was also found to be associated with the increased risk of asthma in children under the recessive model (TT vs. TC + CC: OR = 1.472, 95% CI = 1.067–2.030, *P* = 0.019) and the allele model (T vs. C: OR = 1.320, 95% CI = 1.016–1.713, *P* = 0.037) (Fig. 4). While, no increased or reduced risk of asthma was detected in children under other genetic models (dominant model, TT + TC vs. CC: OR = 1.101, 95% CI = 0.629–1.927, *P* = 0.737; codominant model, TC vs. CC: OR = 0.903, 95% CI = 0.485–1.681, *P* = 0.748 and homozygote model, TT vs. CC: OR = 1.217, 95% CI = 0.679–2.184, *P* = 0.510). We could not perform subgroup analysis in adult group duo to insufficiency of relevant studies included.

### Sensitivity analysis

In order to examine the stability of the pooled ORs, sensitivity analysis was performed by sequentially deleting one study at a time in all of the genetic models. For CHI3L1 rs4950928 and CHIA rs10494132 polymorphisms, the corresponding pooled ORs did not impact in all genetic models, indicating that the results of this meta-analysis were stable.

### Publication bias

**Fig. 5:**
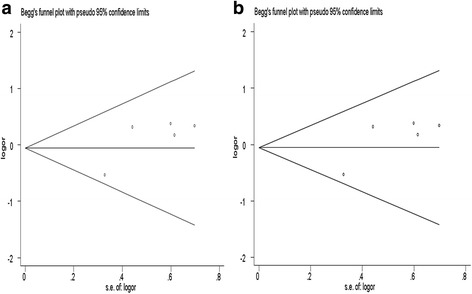
Begg’s funnel plots of publication bias for the association between CHI3L1 rs4950928 (**a**) and CHIA rs10494132 (**b**) polymorphisms and asthma risk under the recessive models (GG vs. GC + CC; TT vs. TC + CC)

Publication bias was evaluated by Begg’s funnel plot and Egger’s regression test. All the shapes of the funnel plots were found to be symmetrical (Fig. 5), indicating that there was no significant publication bias for the associations of CHI3L1 rs4950928 polymorphism and CHIA rs10494132 polymorphism in all the genetic models (all *P* > 0.05) (Table 4).

**Table 4:** Results of publication bias test

Subgroup	Genetic model	Genotype/Allele	Begg’s test		Egger’s test	
			z value	*P *value	t value	*P* value
rs4950928						
Overall	Recessive model	GG vs. GC + CC	0.000	1.000	2.450	0.092
	Dominant model	GG + GC vs. CC	0.490	0.624	0.180	0.866
	Codominant model	GCvs.CC	0.490	0.624	0.060	0.958
	Homozygote model	GG vs. CC	0.980	0.327	1.880	0.157
	Allele model	Gvs.C	0.490	0.624	0.440	0.690
rs10494132						
Overall	Recessive model	TTvs. TC + CC	0.240	0.806	0.770	0.500
	Dominant model	TT + TCvs. CC	0.980	0.327	1.750	0.178
	Codominant model	TCvs. CC	0.490	0.624	1.050	0.373
	Homozygote model	TT vs. CC	1.470	0.142	2.200	0.116
	Allele model	T vs. C	0.490	0.624	1.200	0.317

## Discussion

In the present study, our results indicated that there was no significant association between CHI3L1 rs4950928 polymorphism and asthma susceptibility in overall analyses. However, following subgroup analysis by ethnicity, there was a protective effect of CHI3L1 rs4950928 polymorphism on asthma risk in Caucasians. The G allele of CHI3L1 rs4950928 might be a protective factor against the development of asthma. As for association between CHIA rs10494132 polymorphism and asthma risk, no evidence was found in the overall population, while a significantly increased risk of asthma was found in Asians as well as in children of all ethnicities.

The gene CHI3L1, 10 exons long and located on chromosome 1q31-q32, codes for the chitinase-like protein YKL-40, which is a 40 kDa protein produced by cells in the airway mucosa, such as monocytes, macrophages and neutrophils [10, 17, 25]. YKL-40 binds with ubiquitously expressed chitin, but unlike chitinases CHIT1 and CHIA, which lacks measurable enzymatic chitinase activity [10]. Studies have shown that serum YKL-40 level is increased in patients with asthma and correlated with asthma severity, thickness of the subepithelial basement membrane, and pulmonary function, indicating that serum YKL-40 is an inflammatory biomarker associated with disease activity and mortality in asthmatic patients [12, 26]. Further study has shown that YKL-40 regulates TH2 immune response promoting airway inflammation in asthmatic mouse models as well as actives Akt, Erk, and p38 signaling pathway to promote human bronchial smooth muscle cell proliferation and migration, suggesting that YKL-40 is associated with the pathogenesis of asthma [27]. In addition, Genetic studies have demonstrated that the polymorphic variants of CHI3L1 gene contribute to the pathogenesis of asthma through influencing on airway inflammation and airway remodeling in the asthmatic patients [15, 26]. Recently, the relationship between CHI3L1 rs4950928 polymorphism and susceptibility to asthma was increasingly investigated [7, 18, 20, 21]. A Genome-wide Association Study (GWAS) reported by Ober et al. identified that the G allele of CHI3L1 rs4950928 is protective against asthma in three populations of European ancestry with mild asthma [18]. Another study have demonstrated that an association between CHI3L1 rs4950928 polymorphism and asthma is found, although the risk allele was opposite of that reported by Ober [21]. In contrast, additional publications have shown no significant association between genetic variation in the CHI3L1 rs4950928 and asthma risk [7, 20]. To date, the effect of genetic variation in CHI3L1 on asthma risk has not been fully addressed. Therefore, our meta-analysis provided a more statistical estimation demonstrating the relationship between CHI3L1 rs4950928 polymorphism and risk of asthma.

Our meta-analysis synthesized a total of 1062 cases and 1034 controls from 5 eligible studies to analyze the association between CHI3L1 rs4950928 polymorphism and asthma risk. The pooled results indicated no significant association between CHI3L1 rs4950928 polymorphism and asthma susceptibility. After stratified population by ethnicity, analysis indicated that the G allele of CHI3L1 rs4950928 had a reduced risk for asthma in Caucasians. No subgroup analysis was performed in Asians and Africans due to only 1 study included in Asians and Africans. When stratified population by age, the results remained non-significant in children. We could not perform analysis in adults since only one study was included.

The gene for CHIA is present on chromosome 1q13.1–21.3 and produces acid mammalian chitinase, which is a 50 kDa acid-stable protein [13]. Study has observed that serum CHIA level is overexpressed in ovalbumin-induced mouse model of asthma [16]. In addition, inhibition of CHIA expression reduced TH2-dependent airway inflammation, bronchial hyper-reactivity, and eosinophil counts in these asthma mouse model [16]. In humans, the expression of CHIA is strongly upregulated in lungs of asthmatic patients [16]. Furthermore, the CHIA enzyme has been found to be a downstream protein of interleukin-13, a cytokine implicated in the effect of human asthma [16]. Therefore, the CHIA enzyme has been suggested to be a positive mediator involved in the pathogenesis of asthma. Whereas, conflicting findings regarding its physiological role in asthma have reported that CHIA can inhibit chitin-induced allergic innate immune response in a mouse model by inhibiting eosinophil and basophil recruitment to the lungs [28]. Recently, one genetic study has described exonic CHIA polymorphisms are associated with bronchial asthma in a German pediatric population, indicating that exonic CHIA gene may be under the control of regulatory regions [19]. One promoter SNP, rs10494132, has been reported to be associated with pediatric asthma in Chinese population [23]. However, another three studies has demonstrated that no relationship between CHIA rs10494132 variation and risk of asthma is found [8, 22, 24]. Here we have performed meta-analysis to examine its association with asthma risk.

In this meta-analysis, 796 cases and 744 controls in 4 published articles were included. Results suggested that there was no significant relationship between CHIA rs10494132 polymorphism and asthma susceptibility. When population was stratified based on ethnicity, a positive correlation between CHIA rs10494132 polymorphism and asthma risk was found in Asians. No subgroup analysis was performed in Caucasians due to limited studies included. Moreover, in the subgroup analysis conducted according to age, CHIA rs10494132 variant was also found to be associated with the increased risk of asthma in children. We could not perform subgroup analysis in adult group duo to insufficiency of relevant studies included.

There are several limitations in this meta-analysis. Firstly, sample size in this meta-analysis was small, which might result in bias of the results when evaluating the association of CHI3L1 and CHIA gene polymorphisms with susceptibility to asthma. Secondly, the original data in some studies was lacking, which might limit sufficient statistical power to evaluate the potential effects of gene-gene and gene-environment interactions on the development of asthma. Thirdly, significant between-study heterogeneity was found in some pooled analyses. The exact sources of heterogeneity by meta-regression analysis were unable to further identify because of limited relevant data provided. Subgroup analysis was further performed based on ethnicity and age. However, heterogeneity was not resolved, indicating that other potentially relevant factors such as gender, genotyping method, phenotype of the disease may account for the heterogeneity. Fourthly, asthma is a heterogeneous disease with various genotypes and phenotypes. Although a genetic basis for asthma is undeniable, only a small proportion of heritability can be explained by the previously identified genetic polymorphisms associated with asthma because of the variability of the clinical phenotype. At last, only published studies were retrieved in the meta-analysis and possible publication bias might exist, despite no statistically significant publication bias was detected by Begg’s test or Egger’s test.

## Conclusion

In conclusion, the present meta-analysis demonstrates that there was no significant association between CHI3L1 rs4950928 polymorphism and asthma susceptibility in overall analyses. However, following subgroup analysis by ethnicity, there was a protective effect of CHI3L1 rs4950928 polymorphism on asthma risk in Caucasians. The G allele of CHI3L1 rs4950928 might be a protective factor against the development of asthma. As for association between CHIA rs10494132 polymorphism and asthma risk, no evidence was found in the overall population, while a significantly increased risk of asthma was found in Asians as well as in children of all ethnicities.

## Abbreviations

AMCase or CHIA: Acidic mammalian chitinase
CHI3L1: Chitinase 3–like 1
CHIT: Chitotriosidase
CI: Confidence interval
CNKI: China National Knowledge Infrastructure
GWAS: Genome-wide Association Study
HWE: Hardy-Weinberg equilibrium
IL-17F: Interleukin-17F
OR: Odds ratio
TLR4: Toll-like receptor 4
